# Effects of cold therapy on ecchymosis and pain after subcutaneous heparin injection: a meta-analysis and systematic review

**DOI:** 10.3389/fpain.2026.1853710

**Published:** 2026-08-10

**Authors:** Xiao Min Cao, Xi Xi Feng, Yin Qi Tan, Dan Dan Chen, Xue Sen Zhang

**Affiliations:** 1 School of Medicine, Hang Zhou City University, Hang Zhou, Zhe Jiang, China; 2 First People’s Hospital of Hangzhou Lin'an District, Hangzhou, Zhejiang, China; 3 Hangzhou Lin'an Traditional Chinese Medicine Hospital, Affiliated Hospital, Hangzhou City University, Hangzhou, China

**Keywords:** bruising, cold therapy, ecchymosis, injections, low-molecular-weight heparin, pain management

## Abstract

**Objective:**

This systematic review and meta-analysis aimed to compare the efficacy of three local physical cooling techniques (ice pack compression, vapor cooling spray, and pre-cooled needles) on pain and ecchymosis after subcutaneous anticoagulant injection, re-examine prior controversies with the latest evidence, and provide evidence-based references for clinical nursing.

**Methods:**

Five electronic databases (PubMed, Embase, Cochrane Library, Web of Science, CINAHL) were systematically searched for randomized controlled trials (RCTs) comparing local cold therapy with routine care. Primary outcomes included post-injection pain intensity, ecchymosis incidence, and ecchymosis size.

**Results:**

Ten randomized trials, including seven parallel-group and three randomized within-participant trials, enrolled 807 unique participants. Local cold therapy reduced immediate pain intensity after heparin injection (SMD = −0.64, 95% CI: −0.81 to −0.47, *P* < 0.00001). The analgesic effects did not differ significantly among cooling modalities (*P* = 0.65). Cold therapy increased the odds of being ecchymosis-free at 48 h (OR = 2.77, 95% CI: 1.57–4.88, *P* = 0.0004) and reduced ecchymosis size at 48 h (SMD = −3.03, 95% CI: −4.95 to −1.10, *P* = 0.002). At 72 h, cold therapy increased the odds of being ecchymosis-free (OR = 4.17, 95% CI: 1.31–13.29, *P* = 0.02), but did not significantly reduce ecchymosis size (SMD = −0.76, 95% CI: −1.89 to 0.37, *P* = 0.19). Heterogeneity was substantial for ecchymosis size at 48 h (I2 = 98%); therefore, the magnitude of this pooled effect should be interpreted cautiously.

**Conclusion:**

Local cold therapy may reduce immediate injection pain and early ecchymosis after subcutaneous heparin injection. Evidence at 72 h was outcome-dependent: ecchymosis-free status favored cold therapy, whereas ecchymosis size did not differ significantly. The findings should be interpreted cautiously because of high heterogeneity, outcome-specific risk of bias, and inconsistent reporting of intervention and measurement procedures.

**Systematic Review Registration:**

https://www.crd.york.ac.uk/PROSPERO/view/CRD420261278725, identifier CRD420261278725.

## 1 Introduction

Venous thromboembolism (VTE) is the third most common cardiovascular disease worldwide, characterized by high incidence, disability, and mortality rates, and represents a frequent and severe complication among hospitalized patients. Epidemiological data indicate that the incidence of VTE in hospitalized patients is 1–2 per 1,000 person-years, with significantly elevated risk among critically ill patients, surgical patients, and those with malignant tumors (1).

Subcutaneous administration of low-molecular-weight heparin (LMWH) has become the internationally recognized first-line standard regimen for the prevention and treatment of VTE due to its stable pharmacokinetic profile, convenient administration, no requirement for routine coagulation monitoring, and low bleeding risk, and is widely applied in clinical practice (2, 3). However, owing to the inherent anticoagulant properties of the drug, as well as multiple clinical factors including injection site selection, injection speed, needle gauge, local compression method and duration, patients frequently experience local adverse reactions such as injection site pain, subcutaneous hemorrhage (ecchymosis), induration, and hematoma after injection (4, 5). Multiple studies have reported that the incidence of injection-site ecchymosis following subcutaneous LMWH or unfractionated heparin administration ranges from 60% to 88%. These adverse reactions not only cause physical discomfort and trigger negative emotions such as injection phobia and anxiety, but also reduce patients' trust in clinical nursing care, even leading to refusal of anticoagulant therapy, thereby further increasing the difficulty and workload of clinical nursing (5, 6).

To mitigate local adverse reactions induced by subcutaneous LMWH injection, various non-pharmacological interventions have been explored and implemented clinically. Among these, local cold therapy has become a widely recommended intervention due to its simplicity and low cost. Its core physiological mechanisms can be summarized into two pathways: first, cooling reduces local tissue temperature, inducing constriction of capillary smooth muscle, thereby reducing local extravasation of blood and interstitial fluid and lowering the risk of subcutaneous hemorrhage and ecchymosis (7); second, it slows signal conduction in peripheral nerve endings and elevates the pain threshold, exerting an immediate analgesic effect (8).

Nevertheless, consistent conclusions regarding the exact efficacy of local cold therapy in this clinical setting have not been reached in current evidence-based medicine, with marked inconsistencies across studies. A 2020 meta-analysis by Wang et al. (9) demonstrated that cold application significantly reduced post-injection pain intensity, decreased ecchymosis incidence, and diminished ecchymosis area. In contrast, a meta-analysis published in the same year by Mohammady and Sadeghi (10) reported contradictory findings, suggesting that although cold therapy relieved post-injection pain to some extent, it showed no statistically significant effect on ecchymosis incidence or area. Mohammady proposed in his study that such heterogeneity and controversy may result from differences in study design and the relatively narrow range of interventions included in previous analyses—most studies focused only on traditional cold modalities such as ice packs and cold gel pads, without fully considering variations among different cooling media and their potential impacts on outcomes, including penetration depth and duration of action.

In recent years, with advances in clinical nursing and physical interventions, several novel local cooling techniques have been gradually introduced to alleviate local complications including pain, ecchymosis, and hematoma caused by subcutaneous injection. Innovative cooling modalities such as vapor cooling spray and pre-cooled needles have emerged as research hotspots. Vapor cooling spray achieves rapid cooling through rapid vaporization of liquid media, providing fast onset but relatively superficial tissue penetration. Pre-cooled needle technology delivers targeted cooling to deep tissues along the puncture tract using a pre-chilled needle, offering advantages including no additional preoperative preparation, precise localization, and strong targeting effects. A recent randomized controlled trial published by Kaplan et al. in 2025 showed that pre-cooled needles exhibited favorable efficacy in relieving injection-site pain and preventing subcutaneous ecchymosis (11, 12).

Accordingly, the present study aims to systematically retrieve and include recent high-quality randomized controlled trials covering three categories of cold therapy: conventional cold compression, vapor cooling spray, and pre-cooled needles, to update the existing evidence in this field. Subgroup analyses stratified by cooling technique will be performed to systematically evaluate and compare the effects of different local cold modalities on pain and ecchymosis following subcutaneous heparin injection. By clarifying potential differences in efficacy among cooling methods, this study intends to provide precise and reliable evidence-based support for optimizing nursing strategies during anticoagulant injections in clinical practice.

## 2 Methods

Only randomized controlled trials and quasi-randomized controlled trials were included in this study, while non-randomized controlled studies and other experimental designs were excluded.

Protocol registration: The protocol for this systematic review and meta-analysis was registered in PROSPERO before manuscript preparation (registration number: CRD420261278725).During preparation of the final manuscript, minor methodological refinements were made relative to the initial registered protocol to reflect the available evidence and to improve the transparency of outcome-specific synthesis. These refinements did not change the core review question, population, intervention, comparator, primary outcomes, or the overall interpretation of the findings. All eligibility criteria, outcomes, subgroup analyses, sensitivity analyses, and deviations from the registered protocol are explicitly reported in the Methods section to ensure transparency and reproducibility.

### 2.1 Inclusion and exclusion criteria

Two independent reviewers (XXF and XMC) screened the relevant literature. Inclusion and exclusion criteria were formulated based on the PICOS framework, as detailed below.

P (Population):Inclusion: Adult patients (>18 years old) receiving subcutaneous anticoagulant injection (heparin/low-molecular-weight heparin, LMWH).Exclusion: Patients receiving other anticoagulants during treatment; patients with coagulation disorders (e.g., hemophilia); children and pregnant women (to minimize interference from physiological bleeding tendency).

I (Intervention):Inclusion: Any form of local physical cooling, including pre-cooled needles, cooling spray, and other cold application methods. The intervention group received cold therapy for 2–20 min before or after subcutaneous LMWH injection. Exclusion: Studies combining local cooling with pharmaceutical agents or other physical interventions (e.g., aloe vera, magnesium sulfate wet compress).

C (Comparison):Inclusion: Routine care, placebo, room-temperature needles, or no intervention, with identical injection techniques applied in both intervention and control groups.Exclusion: Studies comparing cold therapy with other active agents (e.g., topical analgesic ointments), unless a blank control group was included in a three-arm design.

O (Outcome):Inclusion: Pain intensity, pain incidence, ecchymosis incidence, and ecchymosis size (maximum diameter or area of ecchymosis).Exclusion: Studies reporting only subjective satisfaction without objective indicators of pain and ecchymosis.

S (Study Design): Inclusion: randomized controlled trials, quasi-randomized controlled trials, or controlled pre-post studies evaluating local physical cooling for subcutaneous heparin/LMWH injection. Exclusion: non-controlled observational studies, reviews, case reports, and studies without extractable pain or ecchymosis outcomes.

### 2.2 Search strategy

We systematically searched PubMed, Embase, Cochrane Library, Web of Science, and CINAHL databases for studies published from January 2015–2025 (detailed search strategies are presented in Supplementary Appendix Table S1). The publication window from 2015 to 2025 was used to define the scope of this review as an updated evidence synthesis rather than as a surrogate marker of study quality. We acknowledge that the methodological quality of a trial depends on its design, conduct, risk of bias, and reporting quality, not on publication year alone. Therefore, all included studies were independently assessed using the Cochrane RoB 2.0 tool regardless of publication year. The 2015–2025 window was selected because previous meta-analyses published in 2020 reached inconsistent conclusions, and several newer cold therapy modalities, including vapocoolant spray and pre-cooled needles, have emerged in recent years. This restriction was intended to capture contemporary injection protocols, nursing practice, and updated intervention modalities. Nevertheless, we recognize that restricting the search to the most recent decade may have excluded earlier relevant evidence; this issue is therefore discussed as a limitation. Two eligible studies were added outside the database retrieval: Buldak et al. (13), identified through manual journal/reference searching, and Şendir et al. (14), identified through reference checking of earlier reviews (13, 14). No other included study was added by manual search. All records were imported into EndNote X9 for screening; duplicates and invalid records were removed. Two reviewers (XXF and XMC) independently screened titles and abstracts for eligibility. Full texts of potentially eligible studies were then evaluated. Any disagreements were resolved through discussion with a third reviewer (YQT).

### 2.3 Data extraction

Two reviewers independently extracted data using a standardized data extraction form; discrepancies were resolved by consensus with a third reviewer. The following data were extracted: first author, journal, publication year, country, study design, inclusion/exclusion criteria, sample sizes of intervention and control groups, participant characteristics, intervention method and duration, injection site, drug name and dosage, and outcome measures (pain intensity score, ecchymosis incidence, ecchymosis size).

### 2.4 Literature search results

A total of 1163 relevant records were initially retrieved from the electronic databases. In addition, two records were identified through manual search/reference checking: Buldak et al. (13) and Şendir et al. (13, 14). All records were imported into EndNote X9, and 244 duplicates were removed. Two independent reviewers screened the titles and abstracts, excluding 889 irrelevant records. Three further records were unavailable for full-text access.

Subsequently, 27 full-text articles were thoroughly evaluated. Among them, 3 studies were excluded because they were published before 2015 (6, 15, 16). This date restriction was intended to update earlier reviews and to reduce clinical and methodological heterogeneity caused by older injection devices, cooling materials, and nursing protocols; however, we acknowledge that excluding pre-2015 studies may omit earlier relevant evidence and may affect the comprehensiveness of the review. Ten studies were excluded for irrelevance to the research topic (9, 17–24) 1 study was excluded due to co-intervention with additional medications (25), and 3 full-text articles remained unobtainable despite contact with the authors (26–28).

**Figure 1:**
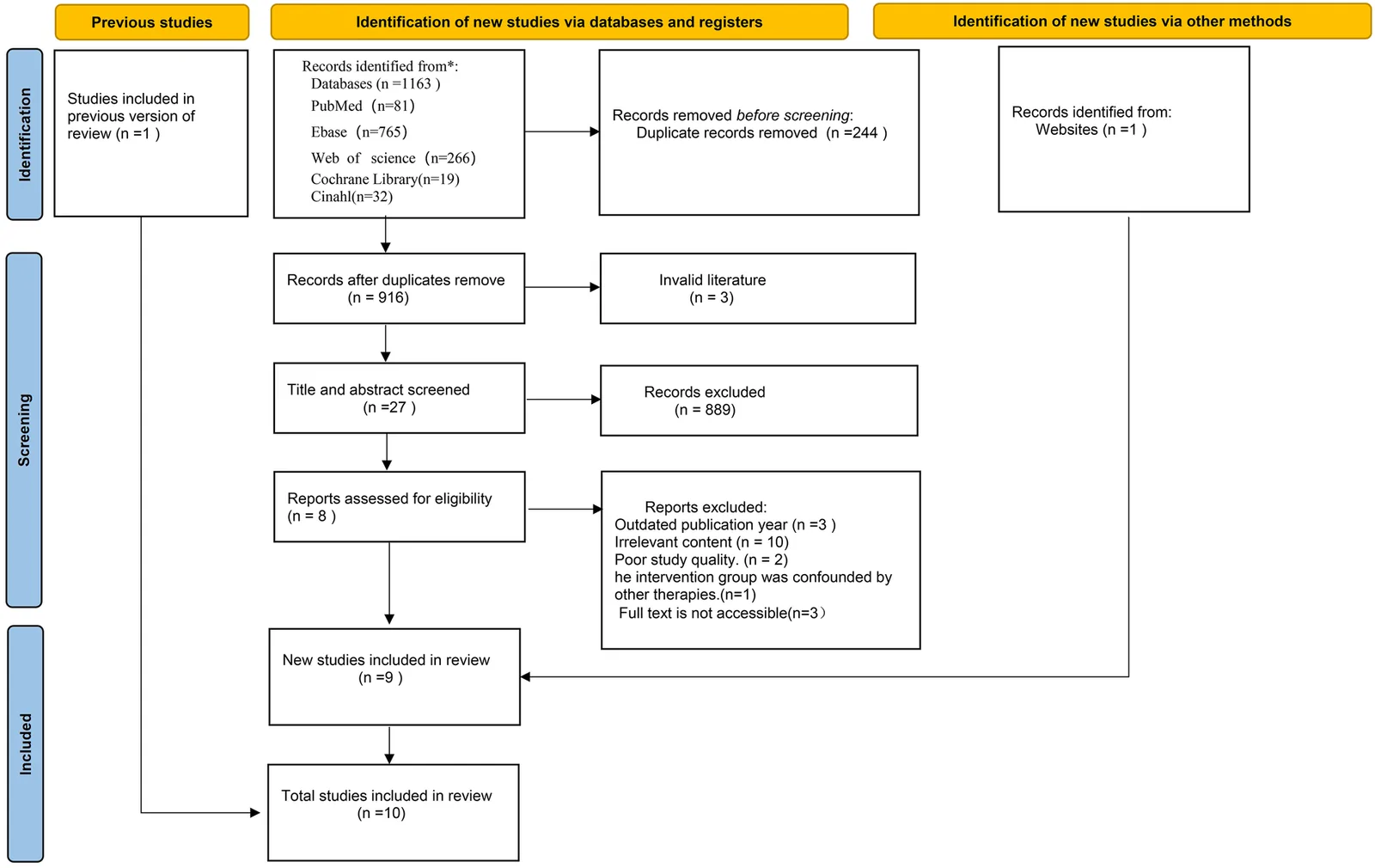
Study flow diagram.

Finally, 10 randomized controlled trials and quasi-randomized controlled trials were included in this study. The detailed study selection process is presented in the PRISMA flow diagram (Figure 1).

### 2.5 Characteristics of included studies

The 10 included studies were conducted in Iran, Turkey, India, and Northern Cyprus, and published between 2015 and 2025. Seven used parallel-group randomization and three used randomized within-participant comparisons. All 10 studies were published in English.

Three cold therapy modalities were investigated: pre-cooled needles (12), conventional cold compression, and cooling spray, with the aim of evaluating their effects on pain and ecchymosis following subcutaneous low-molecular-weight heparin injection. Cold compression included both dry and wet cold application using ice packs or gel packs, with similar underlying mechanisms: local cooling induces vasoconstriction, reduces capillary rupture and hemorrhage, thereby decreasing the incidence and size of ecchymosis and alleviating injection-site pain (9). Similarly, ethyl chloride-based cooling spray used in studies by Ünal et al. and CEVHEROĞLU et al. exerted comparable physiological effects on pain and ecchymosis (29).

The 10 studies enrolled 807 unique participants. Clinical settings included orthopedics, cardiology, neurosurgery, respiratory medicine, and other inpatient departments. Outcome-specific analyses used smaller subsets of studies. In randomized within-participant trials, the same participant contributed observations under more than one treatment condition; therefore, condition-level counts must not be interpreted as additional unique participants. All participants were adults, except that one study did not report an explicit age criterion (30). Regarding anticoagulant agents, 4 studies explicitly used enoxaparin sodium at doses of 60 mg, 40 mg, 4,000 IU, and 6,000 IU, while others only reported low-molecular-weight heparin without specification. Only one study clearly specified a dosage of 4,000 IU (12). One study employed standard prefilled syringes with an air-lock technique (5).The preferred injection site was the periumbilical abdominal region with abundant subcutaneous fat, which is characterized by rapid absorption and mild pain, making it the optimal site for subcutaneous injection (31). Seven studies clearly specified abdominal injection. Only the study by Unal used the arm as the injection site (11), and two studies did not report the injection site.

In the intervention groups, cold therapy was applied before, during, or after heparin injection depending on the protocol. In the pre-cooled needle study, cold stimulation (0–2 °C) was delivered simultaneously with drug administration, providing simultaneous analgesic and anti-ecchymotic effects (12). Several included studies contained multiple intervention arms. Amaniyan's study included a cold compression group and a sequential cold-hot application group to compare their efficacy. CEVHEROĞLU's study included three intervention groups: cold compression, cooling spray, and thermomechanical analgesia (32). Only data relevant to the present research were extracted from these multi-arm studies, with no substantial impact on the overall results (Table 1).

**Table 1:** Characteristics of the included studies.

**Study ID**	**Study Design**	**Age M±SD**	**Participant setting**	**Sample size (E/C)**	**Intervention**		**Outcome**
					**Cold therapy group (E)**	**Control group (C)**	
Shijila (30)	RCT #	Unclear	Patients with stroke, deep vein thrombosis and cardiovascular diseases	25(13/12)	post-injection cold therapy for 3 minutes	Received routine hospital care	a/c
Sharma (22)	RCT #	Unclear	patients with myocardial infarction, acute coronary syndrome, heart failure and other cardiovascular diseases	260(130/130)	post-injection cold therapy for 5 minutes	Received routine hospital care	b/e
Unal (11)	RCT *	45.65±23.51	patients with lower extremity fractures, knee osteoarthritis, ankle fractures and other orthopedic diseases	64(64/64)	Vapocoolant spray applied for 10 seconds immediately before injection	a placebo by a water spray	b/c/d
Amaniyan (33)	RCT #	59.56±1.81	patients with coronary artery disease	120(60/60)	post-injection cold therapy for 20 minutes	Received routine hospital care	b/d
Şendir (14)	RCT #	60.20±10.41	patients who have undergone orthopedic surgery including total hip arthroplasty and total knee arthroplasty	40(20/20)	post-injection cold therapy for 5 minutes	Received routine hospital care	a/b/c
Buldak (13)	RCT #	63.85±9.89	patients following urological surgery	52(26/26)	pre-injection cold therapy for 5 minutes	Received routine hospital care	a/b/d
Simeon (35)	RCT #	Unclear	patients with cardiovascular, neurovascular and other conditions requiring treatment with low-molecular-weight heparin (LMWH)	60(30/30)	post-injection cold therapy for 3 minutes	Received routine hospital care	a/c/e
Cevheroğlu (34)	RCT *	62.31±1.99	patients in internal medicine, cardiology and neurology departments who require subcutaneous injection of low-molecular-weight heparin (LMWH)	54(54/54)	Vapocoolant spray applied at a distance of 15 cm for 5 seconds before injection	Received routine hospital care	a/b/d/e
Karadağ (5)	RCT *	69.1±11.1	patients with chronic obstructive pulmonary disease (COPD) and other conditions requiring subcutaneous injection of heparin	72(72/72)	post-injection cold therapy; 15-second injection duration (exact cold exposure duration was not clearly reported)	Received routine hospital care	a/b/d/e
Kaplan (12)	RCT #	53.43±8.71	patients requiring subcutaneous injection of low-molecular-weight heparin	60(30/30)	injection with needles pre-cooled to 0-2 °C	injection with room-temperature needles	a/b/d

#, parallel-group randomized controlled trial; *, self-controlled before-and-after study; a, pain intensity; b, incidence of bruising; c, size of bruising; d, bruising diameter; e, incidence of pain; SD, standard deviation.

### 2.6 Quality assessment of included studies

**Figure 2:**
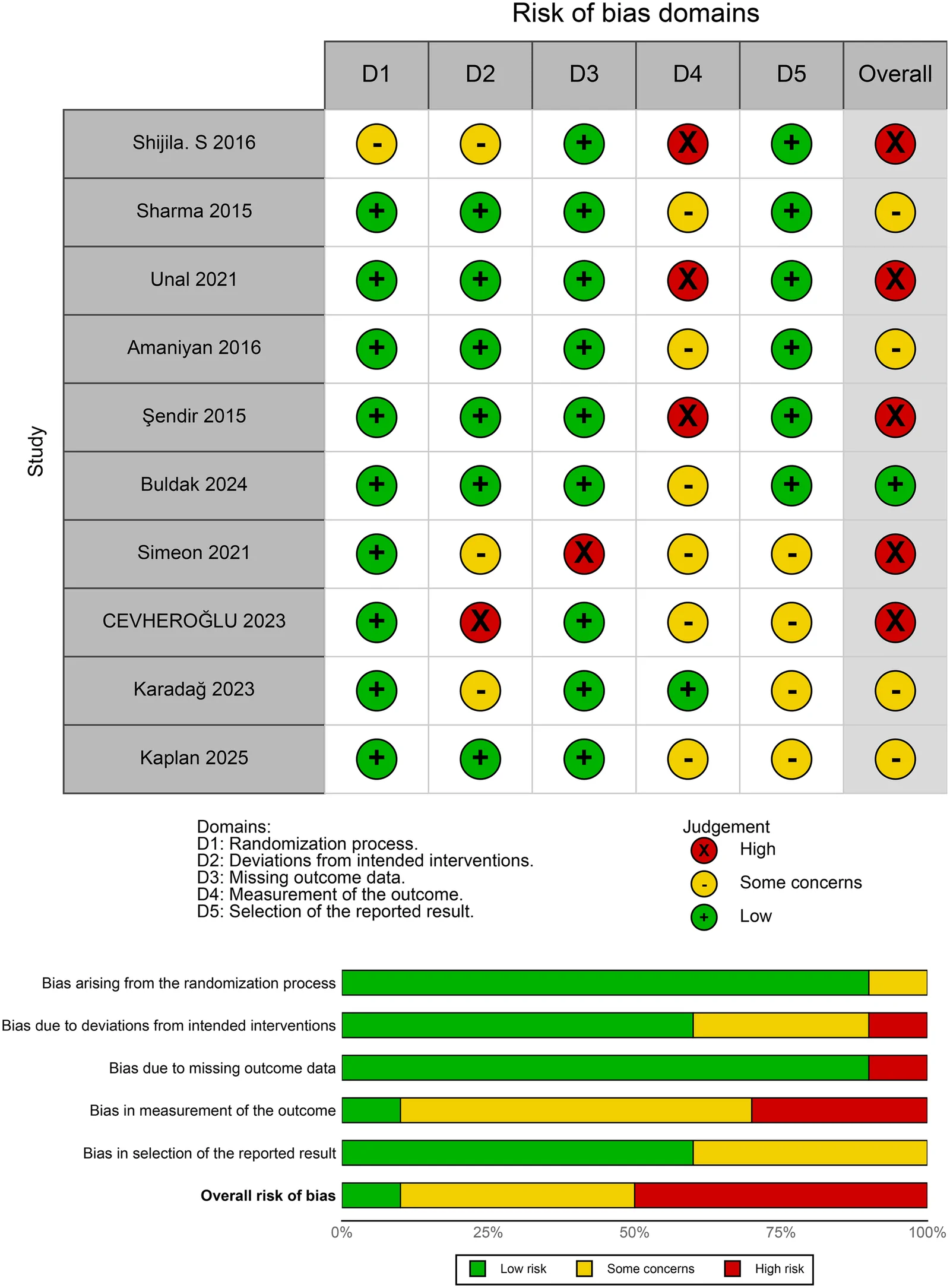
Risk of bias summ.

The risk of bias in included studies was assessed using the Cochrane Risk of Bias tool 2.0 (RoB 2.0). Two reviewers (XXF and XMC) independently evaluated methodological quality and risk of bias; discrepancies were adjudicated by a third reviewer for final judgment. RoB 2.0 comprises six domains: randomization process, deviations from intended interventions, missing outcome data, measurement of the outcome, selection of the reported result, and overall bias. We applied RoB 2.0 at the outcome level for the prespecified primary outcomes, including immediate pain intensity, ecchymosis incidence, and ecchymosis size. Because participant and personnel blinding is practically impossible in cold therapy trials, patient-reported pain was considered particularly vulnerable to performance bias and outcome-measurement bias. Conversely, ecchymosis outcomes can theoretically be assessed by independent or blinded observers; therefore, the “measurement of the outcome” domain was judged separately for pain and ecchymosis whenever sufficient information was available. The overall study-level judgment reported in the bias summary represents the highest risk among relevant outcomes, while the Discussion interprets pain and ecchymosis findings in light of their different susceptibility to bias. The tool is available at: https://www.riskofbias.info/welcome/rob-2-0-tool (Figure 2).

### 2.7 Statistical analysis

All statistical analyses were performed using Review Manager 5.3 software recommended by the Cochrane Collaboration. Data entry and verification were conducted independently by two researchers to ensure accuracy. Before meta-analysis, statistical heterogeneity was quantified using the I² statistic, interpreted according to the Cochrane Handbook: 25%, 50%, and 75% indicating low, moderate, and high heterogeneity, respectively. A fixed-effect model was applied for low heterogeneity (*I*^2^ < 50%), and a random-effect model for high heterogeneity (*I*^2^ ≥ 50%) to provide more conservative effect estimates. For outcome measures:Dichotomous data were analyzed using odds ratios (OR);Continuous data with identical units were analyzed using mean differences (MD);Continuous data with different units were pooled using standardized mean differences (SMD).

For randomized within-participant trials, unique participants were distinguished from condition-level or injection-site observations. Paired effect estimates should be used whenever available. If a primary report provides only condition-level summaries and does not report the within-participant correlation, the resulting approximation should be identified explicitly and examined in sensitivity analyses because treating paired observations as independent may overstate precision.

Statistical significance was set at a two-sided *P* < 0.05.We acknowledge that subgroup analyses stratified by time point for pain and ecchymosis may result in relatively limited studies and sample sizes within each subgroup, potentially reducing statistical power and precision of heterogeneity estimates. Therefore, interpretation of results focused on the clinical relevance of effect sizes and confidence intervals, with caution applied to non-significant findings. Sensitivity analysis was performed using the “leave-one-out” method to assess the influence of individual studies on pooled effect estimates. Furthermore, subgroup analyses were conducted according to the type of cold therapy and the method of ecchymosis measurement.

## 3 Results

### 3.1 Pain intensity

Pain-related outcome data from the included studies were systematically extracted. To evaluate the analgesic effect of cold therapy, multiple time points were assessed in the included studies. Among them, 8 studies reported data on immediate pain intensity after subcutaneous LMWH injection, while 1 study did not report pain-related outcomes; only its ecchymosis-related data were included in the meta-analysis.13 Although the remaining studies reported pain data at multiple time points (4 h, 8 h, 24 h, 48 h, 60 h, and 72 h post-injection), the number of studies eligible for meta-analysis at each time point was insufficient. Therefore, this meta-analysis focused on the effect of cold therapy on immediate pain intensity after subcutaneous LMWH injection to clarify its analgesic efficacy.

In all included studies, pain intensity was evaluated using validated subjective pain rating scales, with the Numerical Rating Scale (NRS) and Visual Analogue Scale (VAS) as the primary tools. Given the differences in scoring dimensions and quantitative criteria among different scales, standardized mean difference (SMD) was selected as the effect size for pooling cross-scale data of the same subjective outcome to evaluate the analgesic effect of cold therapy.

**Figure 3:**
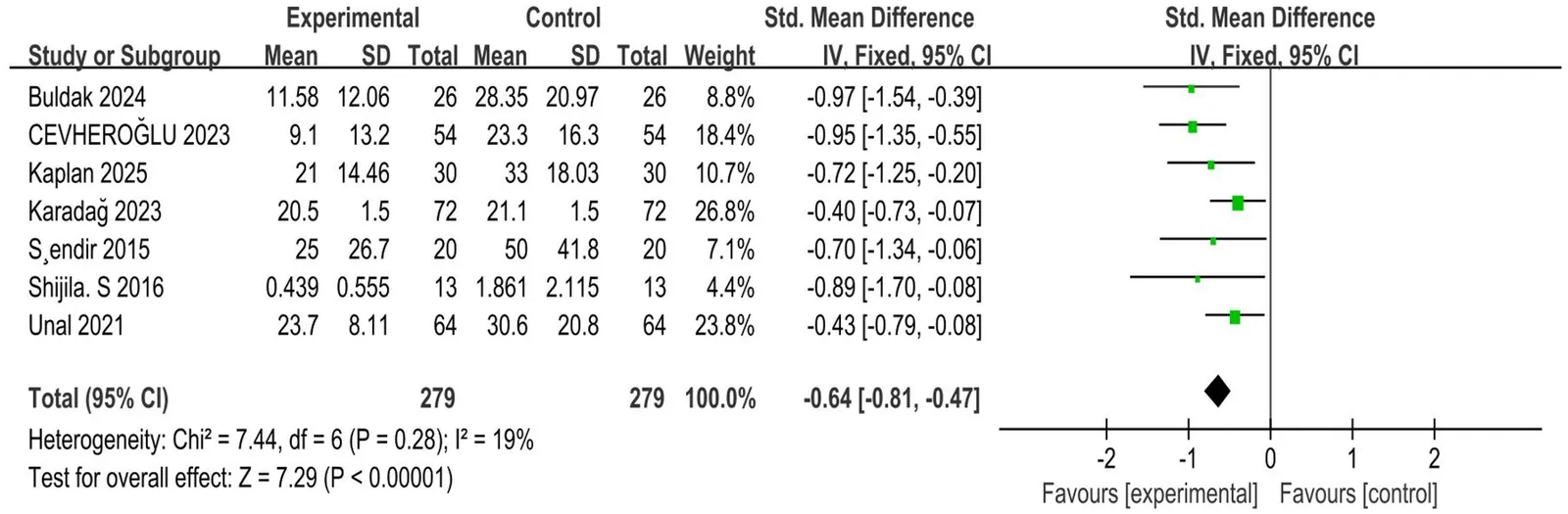
SMD with 95% CI of pain intensity with all cryotherapy techniques (immediate post-injection).

Ultimately, 7 studies were included in the meta-analysis of immediate pain intensity. Heterogeneity testing revealed *χ*^2^ = 7.44, df = 6, *P* = 0.28, and *I*^2^ = 19%, indicating low heterogeneity across included studies (I²<50%). Thus, a fixed-effect model was employed for data pooling. The results showed that, compared with the routine care control group, local cold therapy significantly reduced immediate pain intensity after subcutaneous LMWH injection, with a pooled SMD of −0.64 (95% CI: −0.81 to −0.47, *Z* = 7.29, *P* < 0.00001). The difference between groups was statistically significant (meta-analysis forest plot shown in Figure 3).

### 3.2 Ecchymosis Status

#### 3.2.1 Ecchymosis incidence

Most ecchymosis-related outcomes in the included studies were reported at two time points: 48 h and 72 h after injection. Only 3 studies reported ecchymosis outcomes at 24 h post-injection (12, 33, 34). However, outcome measures were scattered between ecchymosis incidence and ecchymosis size, which precluded pooling of homogeneous effect sizes. One study reported ecchymosis data at 12 h and 60 h (14), and another study did not provide extractable valid ecchymosis data (35). These studies were therefore excluded from the meta-analysis. Accordingly, the main analysis of ecchymosis outcomes focused on 48 h and 72 h after low-molecular-weight heparin injection. Subgroup analyses were predefined according to three cold therapy modalities: cold compression, cooling spray, and pre-cooled needles, to clarify differences in efficacy among various cooling methods.

#### 3.2.2 Ecchymosis incidence at 48 hours post-injection

Seven studies contributed 742 condition-level observations to the 48-hour ecchymosis-incidence analysis (371 intervention-condition and 371 control-condition observations). These do not represent 742 unique participants because several trials used randomized within-participant comparisons. The outcome was the absence of ecchymosis at 48 h. Heterogeneity was moderate (*I*^2^ = 59%); therefore, a random-effects model was used.

**Figure 4:**
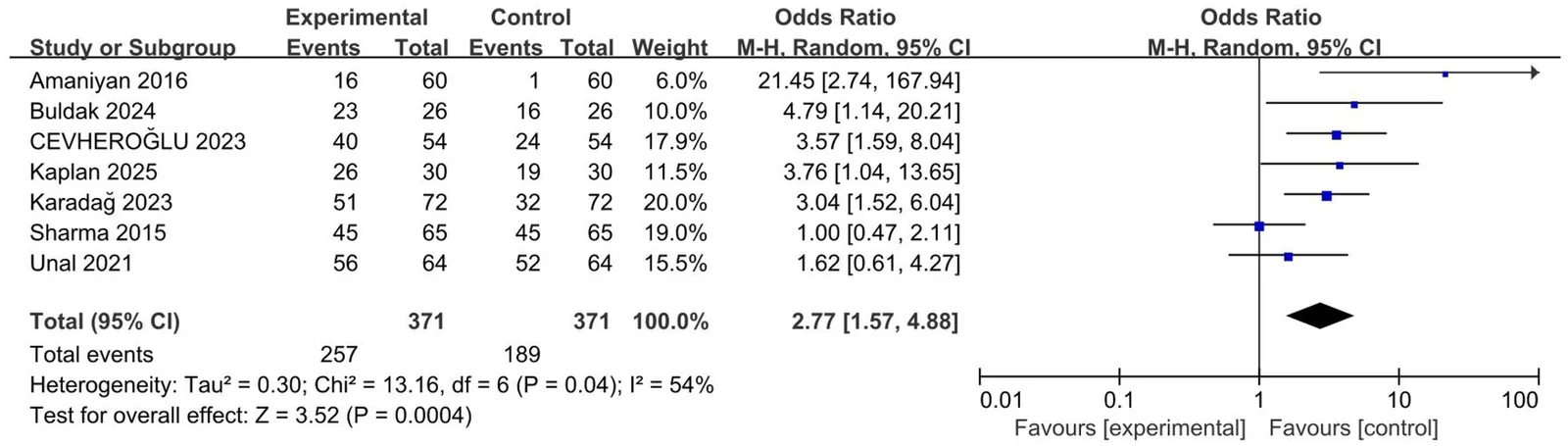
OR with 95% CI of bruising rate with all cryotherapy techniques (At 48 h post-injectio).

The pooled results showed that the probability of being ecchymosis-free at 48 h was significantly higher in the cold therapy group than in the control group, with a pooled odds ratio (OR) of 2.77 (95% CI: 1.57–4.88, *Z* = 3.52, *P* = 0.0004). The difference was statistically significant (forest plot shown in Figure 4).

#### 3.2.3 Ecchymosis incidence at 72 hours post-injection

Five studies contributed 470 condition-level observations to the 72-hour ecchymosis-incidence analysis (235 intervention-condition and 235 control-condition observations). Three studies evaluated conventional cold compression, one evaluated a pre-cooled needle, and one evaluated a cooling spray. The outcome was the absence of ecchymosis at 72 h. These counts are outcome-specific observations rather than the total number of unique participants in the review.

**Figure 5:**
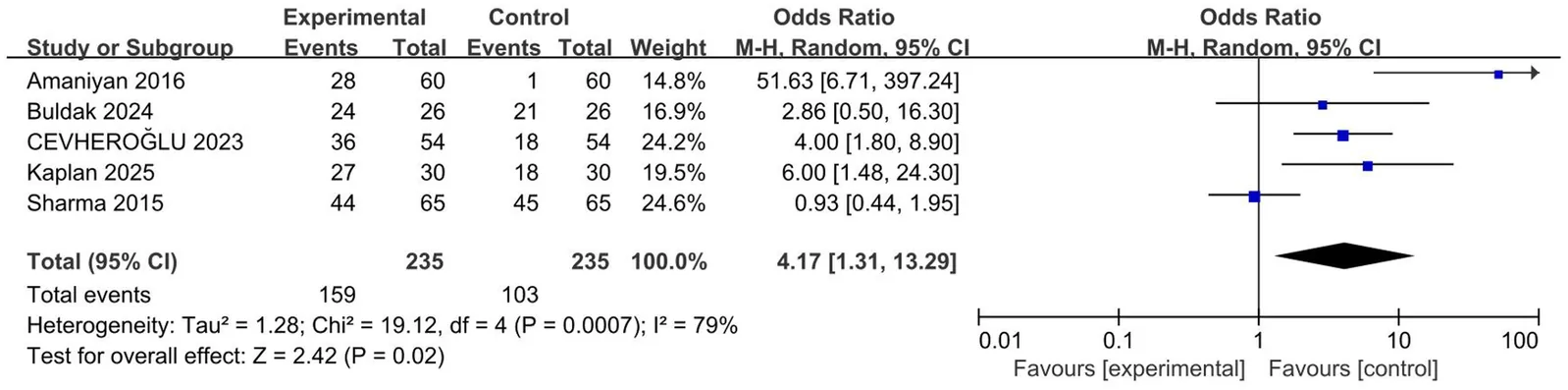
OR with 95% CI of bruising rate with all cryotherapy techniques (At 72 h post-injectio).

Heterogeneity testing demonstrated high heterogeneity (*I*^2^ = 79%, *P* = 0.0007). The Mantel-Haenszel (M-H) random-effect model was therefore applied to pool OR values. The pooled OR was 4.17 (95% CI: 1.31–13.29, *Z* = 2.42, *P* = 0.02), indicating a significantly higher rate of ecchymosis-free status in the cold therapy group. The high heterogeneity across studies suggested that further subgroup analyses were needed to identify sources of heterogeneity (forest plot shown in Figure 5).

### 3.3 Ecchymosis size

We further performed a meta-analysis of data regarding ecchymosis size following subcutaneous low-molecular-weight heparin injection with cold therapy intervention. Based on the distribution of outcome measurement time points in the included studies, data pooling and analysis were conducted at two core time points: 48 h and 72 h post-injection.

\In the included studies, ecchymosis size was mainly measured by either estimated ecchymosis area or maximum ecchymosis diameter. All studies used mm^2^ as the unit for area and mm as the unit for length. Given differences in measurement dimensions and units of the outcome variables, Standardized Mean Difference (SMD) was used as the pooled effect size to evaluate the effect of cold therapy on post-injection ecchymosis size.

#### 3.3.1 Ecchymosis size at 48 hours post-injection

Six studies contributed 524 condition-level observations to the 48-hour ecchymosis-size analysis (262 intervention-condition and 262 control-condition observations). Some observations came from randomized within-participant trials. Heterogeneity was extremely high (*I*^2^ = 98%, *P* < 0.00001); therefore, a random-effects model was used.

**Figure 6:**
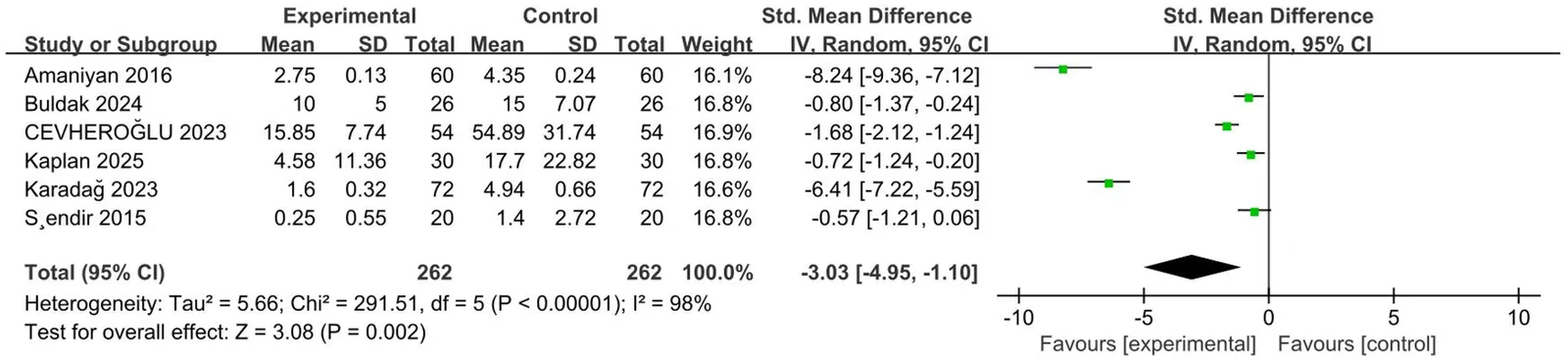
SMD with 95% CI of bruising size as millimeter with all cryotherapy techniques(At 48 h post-injectio).

The primary pooled analysis showed a reduction in ecchymosis size at 48 h (SMD = −3.03, 95% CI: −4.95 to −1.10, *P* = 0.002; Figure 6). The confidence interval remained entirely below zero, so this result was statistically significant. Because heterogeneity was extreme ((*I*^2^ = 98%), the magnitude of the pooled SMD should not be interpreted as a precise clinical effect.(forest plot shown in Figure 6).

#### 3.3.2 Ecchymosis size at 72 hours post-injection

Four studies contributed 260 condition-level observations to the 72-hour ecchymosis-size analysis (130 intervention-condition and 130 control-condition observations) after data from Shijila et al. were excluded as non-extractable. The studies reported either ecchymosis area or maximum diameter. These counts are not the review-wide number of unique participants.

**Figure 7:**
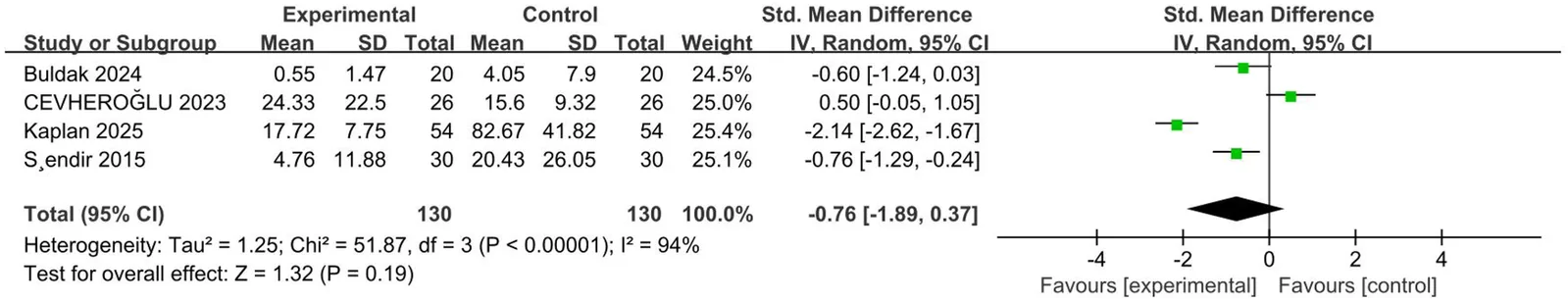
SMD with 95% CI of bruising size as millimeter with all cryotherapy techniques(At 72 h post-injectio).

Owing to extremely high statistical heterogeneity (*I*^2^ = 94%, *P* < 0.00001), SMD was used as the effect size and a random-effect model was applied for data pooling. Pooled results showed that cold therapy did not significantly reduce ecchymosis size at 72 h after LMWH injection, with a pooled SMD of −0.76 (95% CI: −1.89 to 0.37, *P* = 0.19). The difference was not statistically significant (forest plot shown in Figure 7).

### 3.4 Subgroup analyses

#### 3.4.1 Subgroup analysis of different cold therapy modalities on immediate post-injection pain intensity

**Figure 8:**
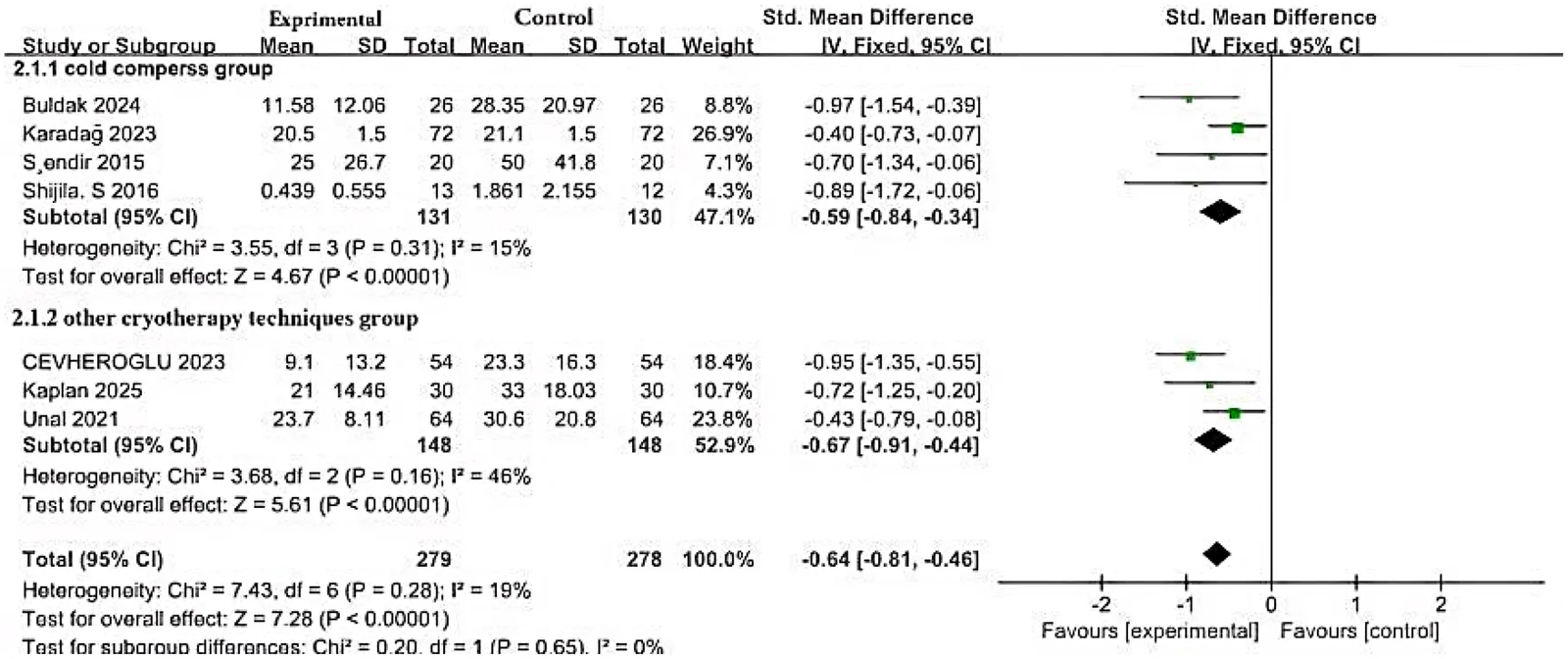
SMD with 95% CI of pain intensity in two groups (immediate post-injection).

Subgroup analysis stratified by cold therapy type was performed for immediate post-injection pain intensity. Results showed that conventional cold compression significantly reduced immediate post-injection pain (SMD = −0.59, 95% CI: −0.84 to −0.34, *P* < 0.00001). Innovative cooling modalities such as cooling spray and pre-cooled needles also exerted significant analgesic effects (SMD = −0.67, 95% CI: −0.91 to −0.44, *P* < 0.00001). No significant difference in effect size was observed between subgroups (*P* = 0.65), indicating comparable analgesic efficacy among the three cold therapy approaches after subcutaneous LMWH injection (forest plot shown in Figure 8).

#### 3.4.2 Subgroup analysis of different cold therapy modalities on ecchymosis incidence

**Figure 9:**
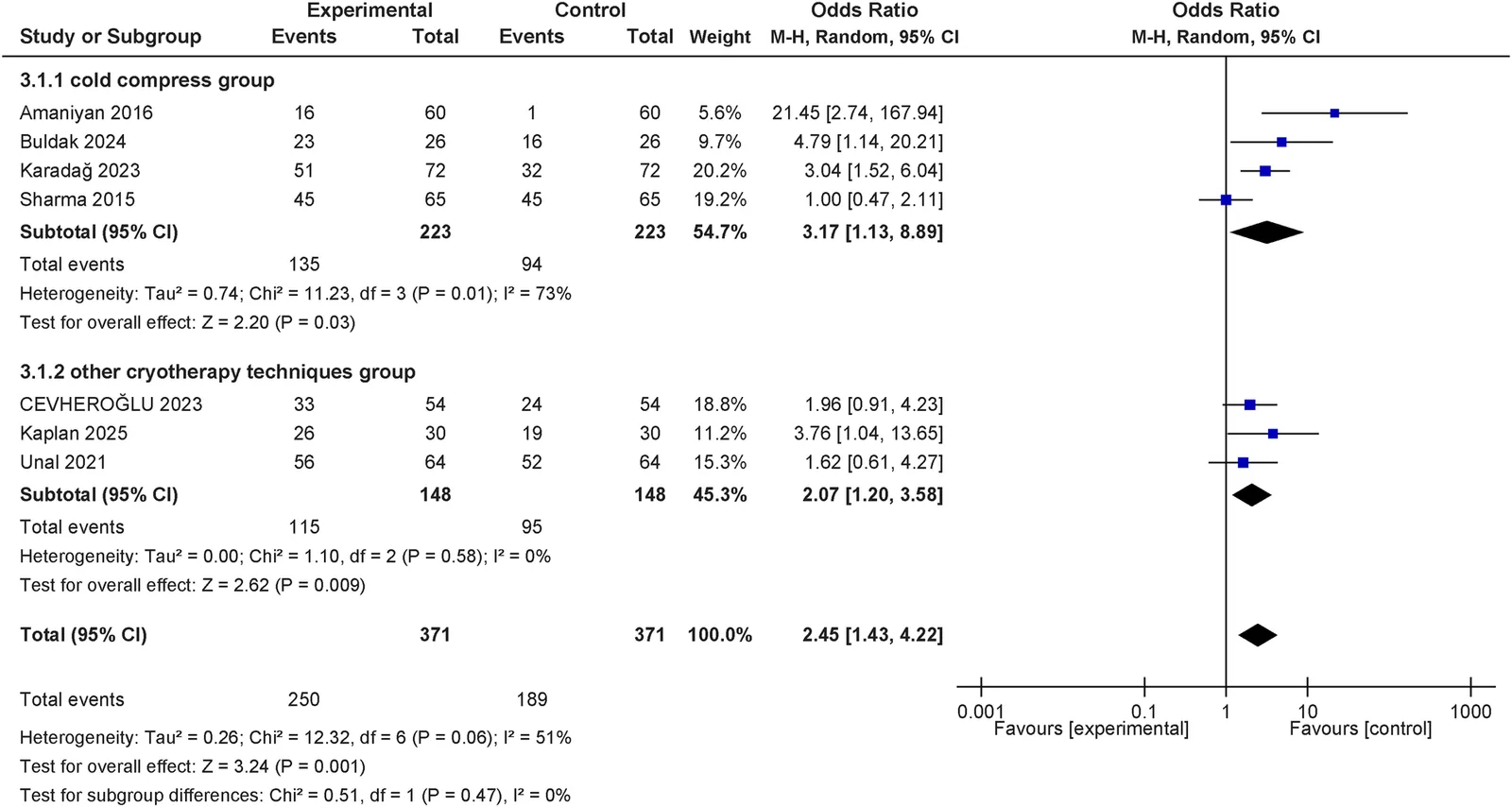
OR with 95% CI of bruising rate in two groups (At 48 h post-injectio).

Subgroup analyses stratified by cold therapy type were further conducted for ecchymosis incidence and ecchymosis size. For ecchymosis-free status at 48 h post-injection, conventional cold compression significantly reduced the risk of ecchymosis (OR = 3.17, 95% CI: 1.13–8.89, *P* = 0.03). Innovative cooling modalities including cooling spray and pre-cooled needles also showed a significant preventive effect (OR = 2.07, 95% CI: 1.20–3.58, *P* = 0.009). No significant between-subgroup difference was detected (*P* = 0.47), suggesting similar efficacy of different cold therapies in reducing ecchymosis risk at 48 h post-injection (forest plot shown in Figure 9).

#### 3.4.3 Exploratory analysis by ecchymosis measurement method

An exploratory stratification considered whether ecchymosis size was reported as maximum diameter or area. However, only one study contributed area data at 48 h, and two diameter-based studies had extreme standardized effects. The subgroup comparison was therefore too sparse and unstable to establish measurement method as the principal cause of heterogeneity. The prespecified primary estimate remains the six-study random-effects result (SMD = −3.03, 95% CI: −4.95 to −1.10, *P* = 0.002; Figure 6).

### 3.5 Sensitivity analysis

To address the extremely high heterogeneity (*I*^2^ = 98%) observed in the meta-analysis of ecchymosis size at 48 h after subcutaneous LMWH injection with cold therapy, a leave-one-out sensitivity analysis was performed by omitting one study at a time to verify the robustness of the core findings.

**Figure 10:**
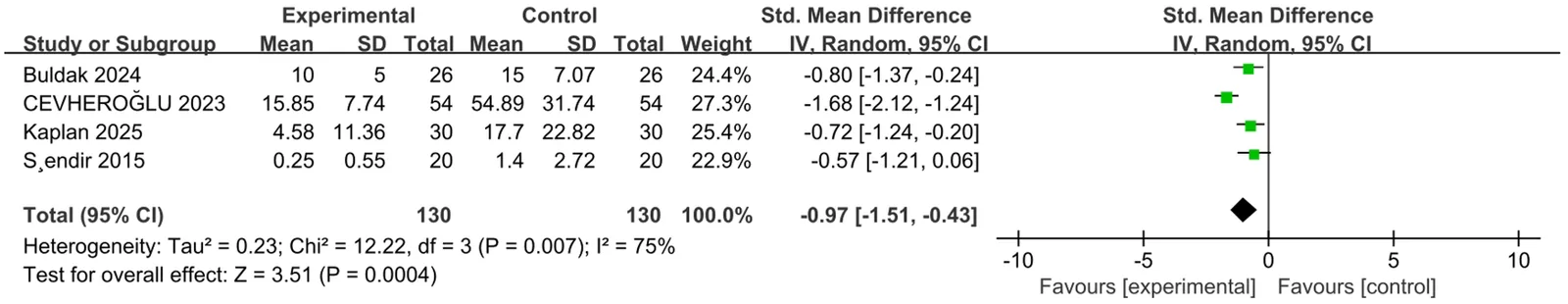
Sensitivity analysis of SMD with 95% CI of bruising size as millimeter with all cryotherapy techniques(At 48 h post-injectio.

The results showed that after excluding the two studies by Amaniyan et al. and Karadağ et al., the recalculated pooled effect size still indicated that cold therapy significantly reduced ecchymosis size at 48 h post-injection (SMD = −0.97, 95% CI: −1.51 to −0.43, *P* = 0.0004). Meanwhile, heterogeneity across studies decreased from the original 98% to 75%, and no directional reversal of the overall effect size was observed, suggesting that the core conclusions of the initial meta-analysis were robust and reliable (forest plot shown in Figure 10).

## 4 Discussion

Low-molecular-weight heparin exhibits favorable efficacy and safety in the prevention of venous thrombosis (36). However, subcutaneous administration is associated with a high incidence of local adverse reactions, including injection-site pain and ecchymosis. These complications not only cause physical discomfort and negative emotions related to injection, but also significantly reduce patient adherence to long-term anticoagulant therapy, adversely affecting clinical nursing quality and medication safety.

Previous evidence-based studies on local cold therapy for such adverse reactions have yielded inconsistent and controversial conclusions. Furthermore, most studies have focused exclusively on conventional ice pack compression, without providing systematic evaluation or quantitative analysis of novel cooling techniques such as vapor cooling spray and pre-cooled needles. Consequently, clinical nursing practice lacks comprehensive and up-to-date evidence-based support.

Against this background, the present systematic review and meta-analysis included 10 randomized controlled trials with a total of 807 participants. This study aimed to comprehensively evaluate the efficacy of local cold therapy in reducing pain intensity, lowering ecchymosis incidence, and decreasing early ecchymosis area following subcutaneous low-molecular-weight heparin injection. Subgroup analyses were performed to compare the effectiveness of conventional cold therapy and novel cooling modalities on pain relief and ecchymosis prevention. The ultimate goal was to provide updated and comprehensive evidence and practical guidance for non-pharmacological interventions targeting adverse reactions related to heparin injection.

### 4.1 Main findings

The present meta-analysis demonstrated that local cold therapy significantly alleviated immediate pain intensity after subcutaneous low-molecular-weight heparin injection, which is consistent with most previous clinical studies and meta-analyses. Regarding the physiological mechanism of pain relief, relevant systematic reviews by Wang et al. and Mohammady et al. both adopted the gate control theory to explain the analgesic effect of cold therapy: local cold stimulation activates cutaneous receptors, inhibits pain signal transmission to T cells in the dorsal horn of the spinal cord, and regulates glial cell activity to block ascending pain pathways, thereby producing analgesia (9, 10, 37). In all included studies, the duration of cold therapy was limited to 20 min before or after injection, and this time window is reasonable for the clinical management of acute injection-related pain (38, 39). With respect to ecchymosis, two meta-analyses published in 2020 reached markedly divergent conclusions. Wang et al. reported that cold application significantly reduced ecchymosis incidence and area at 72 h post-injection, whereas Mohammady et al. found no statistically significant effect of cold therapy on ecchymosis-related outcomes.

The present study incorporated conventional cold compression, cooling spray, and pre-cooled needles. At 48 h, cold therapy improved both ecchymosis-free status and ecchymosis size. At 72 h, the results differed by outcome: ecchymosis-free status favored cold therapy (OR = 4.17, *P* = 0.02), whereas ecchymosis size did not differ significantly (SMD = −0.76, *P* = 0.19).

Mohammady et al. explicitly stated that the inconsistent conclusions compared with Wang et al. were mainly attributed to differences in literature inclusion criteria and study designs. Specifically, Wang et al. included more non-randomized controlled studies, which may introduce bias into the pooled results.

In another systematic review evaluating the effect of injection speed on pain and ecchymosis during subcutaneous low-molecular-weight heparin administration, Mohammady et al. reported that slow injection slightly relieved pain within 48 h and might reduce ecchymosis incidence and size at 48 and 60 h post-injection. However, the authors emphasized that the certainty of evidence for these findings was very low (40).

Besides measurement methods, the development and progression of ecchymosis vary considerably among individuals and are affected by multiple confounding factors, including injection site, drug dosage, and patient sex. Existing evidence confirms that the incidence of ecchymosis is significantly lower with abdominal injection than with arm injection, and female patients are at higher risk of local skin reactions than males (41). All these confounding factors may serve as potential sources of the high heterogeneity observed across the included studies.

### 4.2 Innovations in subgroup analysis

The present study is among the first systematic review and meta-analysis to perform pre-specified subgroup analyses to comprehensively compare the clinical efficacy of three distinct local cold therapy modalities—conventional ice pack compression, vapor cooling spray, and pre-cooled needles—in relieving pain and preventing ecchymosis following subcutaneous low-molecular-weight heparin injection. Subgroup results demonstrated no statistically significant differences among the three cooling methods in alleviating immediate post-injection pain or reducing ecchymosis incidence at 48 h. These findings indicate that the core clinical benefits of cold therapy are not dependent on a specific cooling medium, and all cold modalities exert favorable effects on pain relief and ecchymosis prevention.

Most previous meta-analyses on this topic focused solely on conventional interventions such as ice packs and cold gel pads, without including innovative cooling techniques including vapor cooling spray and pre-cooled needles that have been increasingly adopted in clinical practice, leading to limitations in evidence coverage. The present study incorporated the latest prospective randomized controlled trial of pre-cooled needles published by Kaplan et al. in 2025, which demonstrated that pre-cooled needles effectively reduced injection pain, improved patient satisfaction, and decreased ecchymosis formation during subcutaneous heparin administration (12). Studies by CEVHEROĞLU and Ünal et al. also confirmed that vapor cooling spray provides favorable effects in alleviating injection-related pain and ecchymosis (11, 34).

However, owing to the limited number of high-quality clinical studies on cooling spray and pre-cooled needles, the sample size available for meta-analysis was relatively small, which somewhat restricts the generalizability of subgroup findings for these two innovative techniques. Further large-sample, multicenter, and rigorously designed randomized controlled trials are warranted to verify their long-term clinical efficacy and application value.

### 4.3 Clinical implications

Based on the meta-analysis results, we propose the following recommendations for the clinical application of the three cold therapy modalities:

First, local cold therapy is recommended as a routine non-pharmacological intervention for adverse reactions following subcutaneous low-molecular-weight heparin injection in adult patients with normal coagulation function and no contraindications to cold application. It can effectively reduce pain, lower the early risk of ecchymosis, and improve treatment adherence and nursing satisfaction. Nevertheless, clinical translation should distinguish between target outcomes. Pre-injection cooling mainly aims to reduce puncture-related pain by lowering cutaneous nerve conduction and pain sensitivity (42), whereas post-injection cooling is more directly related to vasoconstriction and limitation of local blood extravasation (43). Simultaneous cooling, as in pre-cooled needle protocols, may theoretically combine analgesic and anti-ecchymotic effects, but current evidence remains limited.

Second, cold therapy modalities can be flexibly selected based on clinical settings, available resources, and the expected depth and duration of cooling. Conventional ice packs and gel compresses are low-cost and easily accessible, making them suitable for primary hospitals and general wards; however, they require additional application time and careful skin protection (44, 45). Vapor cooling spray provides rapid onset and simple operation, making it suitable for emergency departments and fast-paced outpatient settings, but its cooling effect is relatively superficial and short-lived (42). Pre-cooled needles enable simultaneous injection and intervention without additional waiting time, rendering them potentially suitable for high-frequency heparin administration departments such as cardiology and orthopedics, although more multicenter evidence is needed before routine implementation (46).

Third, standardized cold therapy procedures are strongly recommended. Based on available evidence, a cooling duration of 3–5 min may be considered for conventional cold application, with the timing selected according to the intended outcome: before injection when pain control is prioritized, after injection when ecchymosis control is prioritized, or before-and-after protocols when both outcomes are clinically important. The periumbilical abdominal region remains the preferred injection site in many protocols because it has abundant subcutaneous tissue and is associated with relatively mild pain and stable absorption (4, 45, 47). However, adipose tissue thickness may influence heat transfer: patients with thicker abdominal subcutaneous fat may experience slower cooling of deeper tissues, particularly with short superficial applications such as vapocoolant spray. Therefore, the same cooling duration may not produce identical tissue-temperature changes across patients or anatomical sites. Nurses should select the site and device carefully, avoid excessive cooling pressure or duration, and monitor for local frostbite, nerve irritation, and reduced comfort (39, 48).

Because most primary studies did not provide detailed stratified data by anatomical site, abdominal fat thickness, or cooling-device temperature profile, the above recommendations should be viewed as pragmatic rather than definitive. Future trials should report injection site, subcutaneous fat thickness or body mass index, device temperature, duration of exposure, distance of spray application, and whether cooling was performed before, during, or after injection, so that clinically applicable protocols can be developed.

### 4.4 Limitations of the study

This study has several limitations. First, risk of bias was frequently unclear or high, and susceptibility differed by outcome. Self-reported pain was vulnerable to lack of blinding, whereas ecchymosis measurement depended on assessor blinding and measurement standardization. Second, subgroup analyses for cooling spray and pre-cooled needles included few studies. Third, ecchymosis size at 48 h showed extreme heterogeneity (*I*^2^ = 98%). Differences in measurement metric, cooling duration, injection site, heparin dose, and participant characteristics may all have contributed, but the available subgroup data were insufficient to identify one dominant source. Fourth, three trials used randomized within-participant comparisons. When paired correlations were unavailable, analysis based on condition-level summaries may have overstated precision. Fifth, the current evidence set used a 2015 lower date limit; the unrestricted search and corresponding reanalysis must be completed before resubmission. Primary studies also used inconsistent pain scales and ecchymosis measurement procedures. These differences may have contributed to heterogeneity and reduce comparability across studies. Long-term safety and patient satisfaction were reported inconsistently, which limits assessment of the broader clinical value of cold therapy.

## 5 Conclusions and future research directions

Based on the available evidence, this study indicates that local cold therapy is an effective and easy-to-implement non-pharmacological intervention to reduce immediate pain and early ecchymosis following subcutaneous low-molecular-weight heparin injection. No significant statistical differences were observed among conventional cold compression, vapor cooling spray, and pre-cooled needles in relieving immediate post-injection pain and reducing ecchymosis at 48 h. Clinical nurses may select the appropriate cooling method according to specific clinical settings, target outcomes, available resources, and patient characteristics, while recognizing that current evidence is limited by heterogeneity, outcome-specific bias, and incomplete reporting of intervention parameters.

This study updates the evidence base in this field and provides a reference for resolving previous controversies and guiding clinical nursing practice during heparin injection. In view of the limitations of this study, future large-sample, multicenter, low-bias RCTs are warranted to verify the efficacy and safety of different cold therapy modalities. Further research should also explore the optimal intervention parameters of cold therapy and promote the development of standardized clinical protocols, so as to reduce complications, improve patient satisfaction, and enhance the quality and safety of nursing care.

## Data Availability

The original contributions presented in the study are included in the article/Supplementary Material, further inquiries can be directed to the corresponding author.
