# Supplementary material for: Effects of cold therapy on ecchymosis and pain after subcutaneous heparin injection: a meta-analysis and systematic review
Source: Front Pain Res (Lausanne). 2026 Aug 10;7:1853710. doi: 10.3389/fpain.2026.1853710 (PMC13501458; doi:10.3389/fpain.2026.1853710)
Supplement: Supplementary file 1 [file Table1.docx]

Table.1 search strategies

**PubMed**

| **Search** | **Query** | **Items**  **found** |
| --- | --- | --- |
| 1# | Search  (("Heparin"[Mesh]OR"Heparin,Low-Molecular-Weight"[Mesh] ) OR ( heparin* OR LMWH OR nadroparin* OR fraxiparin* OR enoxaparin* OR Clexane OR klexane OR lovenox OR dalteparin* OR ardeparin* OR normiflo OR tinzaparin* OR logiparin OR Innohep OR certoparin* OR sandoparin OR reviparin* OR clivarin* OR danaparoid ) OR ( Dalteparin OR Liquaemin OR AVE5026 OR op2123 OR parvoparin OR low epa OR lowhepa OR lohela OR lohepa OR fluxum OR parnaparin* OR orgaran OR lomoparan OR kb101 OR calciparin* OR tedegliparin* OR seleparin* OR cy216 OR pk10169 OR emt967 OR emt966 OR tedelparin OR rd11885 OR monoembolex OR embolex OR cy222 OR szibor OR Zibor OR bemiparin* OR ardeparin* OR antixarin OR FR860 OR Kabi2165 OR Fragmin ) ) | 122303 |
| 2# | Search  (("Contusions"[Mesh]OR"Hematoma"[Mesh]OR"Ecchymosis"[ Mesh] OR "Purpura"[Mesh]) OR ( indurat* OR contus* OR  bruis* OR hematoma* OR haematoma* OR suggillation OR  petechiae )) OR ( "Pain"[Mesh] OR pain* OR ache OR aches ) | 1434231 |
| 3# | Search  (("Cryotherapy"[Mesh] OR "Ice"[Mesh]) OR ("cold therapy"  OR cryotherapy OR "cold compress" OR "cold application" OR icing) OR (cold)) | 280667 |
| 4# | #1 AND #2 AND #3 | 81 |

**Ebase**

| **Search** | **Query** | **Items**  **found** |
| --- | --- | --- |

| 1# | liquaemin OR ave5026 OR (op AND 2123) OR parvoparin OR  (low AND epa) OR lowhepa OR lohela OR lohepa OR fluxum  OR orgaran OR lomoparan OR (kb AND 101) OR calciparin OR  tedegliparin OR seleparin OR (Cy AND 216) OR (pk AND  10169) OR (emt AND 967) OR (emt AND 966) OR tedelparin  OR 'rd 11885' OR parnaparin OR (mono AND embolex) OR  monoembolex OR embolex OR 'cy 222' OR szibor OR zibor OR  bemiparin OR antixarin OR fr860 OR kabi2165 OR fragmin  OR heparin OR 'low molecular weight heparin' OR Imwh OR  nadroparin OR fraxiparin OR enoxaparin OR clexane OR klexane OR lovenox OR dalteparin OR ardeparin OR  normiflo OR tinzaparin OR logiparin OR innohep OR certoparin OR sandoparin OR reviparin OR clivarin OR danaproid OR danaparoid | 299, 113 |
| --- | --- | --- |
| 2# | contusion OR indurat OR bruise OR bruising OR hematoma  OR haematoma OR ecchymosis OR suggillation OR purpura OR  petechia OR pain OR aches OR ache | 2290407 |

| 3# | (therapies, AND cold)OR cryotherapies OR(therapy, AND cold) OR (cold AND therapies) OR ice OR icing OR (cold AND therapy) OR cryotherapy OR 'cold  compress' OR (cold AND application) OR cold | 392002 |
| --- | --- | --- |
| 4# | #1 AND #2 AND #3 | 765 |

**Web	of	science**

| **Search** | **Query** | **Items**  **found** |
| --- | --- | --- |
| 1# | TS=(Dalteparin）OR TS=(Liquaemin) OR TS=(AVE5026) OR TS=(op 2123) OR TS=(parvoparin) OR TS=(low epa) OR TS=(lowhepa) OR TS=(lohela) OR TS=(lohepa) OR  TS=(fluxum) OR TS=(parnaparin) OR TS=(organ) OR  TS=(lomoparan) OR TS=(kb 101) OR TS=(calciparin*) OR TS=(tedegliparin*) OR TS=(seleparin*) OR TS=(cy 216) OR TS=(pk 10169) OR TS=(emt 967) OR TS=(emt 966)  OR TS=(tedelparin)ORTS=(rd11885)ORTS=(parmaparin*) OR TS=(monoembolex) OR TS=(monoembolex) OR  TS=(embolex) OR TS=(cy222) OR TS=(szibor) OR  TS=(Zibor) OR TS=(bemiparin*) OR TS=(ardeparin*) OR TS=(antiarin) OR TS=(FR860) OR TS=(Kabi2165 ) OR TS=(Fragmin) OR TS=(Heparin) OR  TS=(Heparin,Low-Molecular-Weight) OR TS=(heparin *) OR TS=(LMWH) OR TS=(nadroparin*) OR  TS=(enoxaparin*) OR TS=(Clexane) OR TS=  (fraxiparin*) OR TS=(lov enox) OR TS=(kl exane) OR TS=(dalteparin*) OR TS=(ardeparin)  OR TS=(normiflo) OR TS=(tinzaparin) OR TS=(logiparin) OR TS=(Innohep) OR TS=  (certoparin) OR TS=(sandoparin) OR TS=(reviparin) OR | 830908 |

|  | TS=(clivarin*) OR TS=(danaproid)  OR TS=(danaparoid) |  |
| --- | --- | --- |
| 2# | TS=(Contusions) OR TS=(indurat) OR TS=(contus*) OR TS= (bruis*) OR TS=(Hematoma) OR TS=(hematoma*) OR TS= (haematoma*) OR TS=(Ecchymosis) OR  TS=(ugllation) OR TS=(Purpura) OR TS=(Petechiae) OR TS=(Pain) OR TS=(Aches) OR TS=(pain*) OR TS=(Ache) | 1243372 |
| 3# | TS=("Cold Therapy") OR TS=("Therapies, Cold") OR  TS=(" Cryotherapies") OR TS=("Therapy, Cold") OR  TS=("Cold Therapies") OR TS=(Ice) OR TS=(ICING) OR TS=("cold therapy" ) OR TS=("Cryotherapy") OR  TS=("cold compress") OR TS=(" cold application") OR TS=(cold) | 738404 |
| 4# | #1 AND #2 AND #3 | 264 |

**Cochrane Library**

| **Search** | **Query** | **Items**  **found** |
| --- | --- | --- |
| #1 | MeSH descriptor: [Heparin] explode all trees | 6191 |
| #2 | (Heparinic Acid OR Unfractionated Heparin OR Heparin, Unfractionated OR Sodium Heparin OR Heparin, Sodium OR Heparin Sodium OR alpha-Heparin OR alpha Heparin OR Liquaemin):ti,ab,kw | 4098 |
| #3 | MeSH descriptor: [Heparin, Low-Molecular-Weight] explode all trees | 2707 |
| #4 | (Heparin, Low Molecular Weight OR Low Molecular Weight Heparin OR Low-Molecular-Weight Heparin OR LMWH):ti,ab,kw | 4627 |

| #5 | ((*heparin*) OR (LMWH) OR (nadroparin*) OR  (fraxiparin*) OR (enoxaparin)):ti,ab,kw | 15497 |
| --- | --- | --- |
| #6 | ((Clexane) OR (klexane) OR (lovenox)OR (dalteparin*) OR (ardeparin)):ti,ab,kw | 1041 |
| #7 | ((Fragmin) OR (Kabi2165) OR (antixarin) OR  (ardeparin*) OR (kb 101) OR (lomoparan) OR (orgaran) OR (parnaparin) OR (fluxum)):ti,ab,kw | 349 |
| #8 | #1 OR #2 OR #3 OR #4 OR #5 OR #6 OR #7 | 15780 |
| #9 | MeSH descriptor: [Pain] explode all trees | 75044 |
| #10 | (Suffering, Physical OR Physical Suffering OR Physical Sufferings OR Sufferings, Physical OR Ache OR Aches OR Pain, Burning OR Burning Pain OR Burning Pains OR Pains, Burning OR Pain, Crushing OR Crushing Pain OR Crushing Pains OR Pains, Crushing OR Pain, Migratory OR Migratory Pain OR Migratory Pains OR Pains, Migratory OR Pain, Radiating OR Pains, Radiating OR Radiating Pain OR Radiating Pains OR Pain, Splitting OR Pains, Splitting OR Splitting Pain OR Splitting Pains):ti,ab,kw | 20971 |
| #11 | ((Ache) OR (Aches) OR (pain*)):ti,ab,kw | 289121 |
| #12 | MeSH descriptor: [Purpura] explode all trees | 673 |
| #13 | (Purpuras OR Petechiae):ti,ab,kw | 2169 |
| #14 | MeSH descriptor: [Ecchymosis] explode all trees | 157 |
| #15 | (Hematomas):ti,ab,kw | 8701 |
| #16 | MeSH descriptor: [Contusions] explode all trees | 170 |
| #17 | #9 OR #10 OR #11 OR #12 OR #13 OR #14 OR #15 OR #16 | 308276 |
| #18 | MeSH descriptor: [Ice] explode all trees | 224 |

| #19 | ((Cold Therapy) OR (Therapies, Cold) OR (Cryotherapies) OR (Therapy, Cold) OR (Cold  Therapies)):ti,ab,kw | 8541 |
| --- | --- | --- |
| #20 | MeSH descriptor: [Cryotherapy] explode all trees | 2338 |
| #21 | (Cryotherapies OR Cold Therapy OR Cold Therapies OR Therapies, Cold OR Therapy, Cold):ti,ab,kw | 8541 |
| #22 | #18 OR #19 OR #20 OR #21 | 9889 |
| #23 | #8 AND #17 AND #22 | 19 |

**CINAHL**

| **Search** | **Query** | **Items**  **found** |
| --- | --- | --- |
| #1 | ( (MH “Heparin, Low-Molecular-Weight+“) OR (MH “Heparin”) OR (“low molecular weight heparin*” OR LMWH OR enoxaparin OR dalteparin OR nadroparin OR tinzaparin OR fondaparinux OR “Lovenox” OR “Clexane” OR “Fragmin”) ) | 189916 |
| #2 | ( (MH “Cold Therapy”) OR (MH “Cryotherapy+“) OR (MH “Ice”) OR (“cold therap*” OR cryotherap* OR “cold application*” OR “ice pack” OR “cold pack” OR “cold compress*” OR cooling) ) | 992609 |
| #3 | ( (MH “Bruises”) OR (MH “Pain”) OR (MH “Hematoma”) OR (MH “Contusions”) OR (MH “Induration”) OR (MH “Purpura”) OR (bruis* OR ecchymos* OR hematoma* OR haematoma* OR “blood blot*” OR pain* OR painful OR discomfort OR indurat* OR swell* OR purpura OR contusion*) ) | 5945085 |
| #4 | #1AND #2 AND #3 | 32 |
